# Differential effects of NOX2 and NOX4 inhibition after rodent spinal cord injury

**DOI:** 10.1371/journal.pone.0281045

**Published:** 2023-03-10

**Authors:** Guzal Khayrullina, Sara Bermudez, Deanna Hopkins, Young Yauger, Kimberly R. Byrnes

**Affiliations:** 1 Anatomy, Physiology and Genetics Department, Uniformed Services University, Bethesda, MD, United States of America; 2 Henry M. Jackson Foundation for the Advancement of Military Medicine, Inc, Bethesda, MD, United States of America; 3 Neuroscience Graduate Program, Uniformed Services University of the Health Sciences, Bethesda, Maryland, United States of America; Amity University Uttar Pradesh, INDIA

## Abstract

Reactive oxygen species (ROS) are a contributing factor to impaired function and pathology after spinal cord injury (SCI). The NADPH oxidase (NOX) enzyme is a key source of ROS; there are several NOX family members, including NOX2 and NOX4, that may play a role in ROS production after SCI. Previously, we showed that a temporary inhibition of NOX2 by intrathecal administration of gp91ds-tat immediately after injury improved recovery in a mouse SCI model. However, chronic inflammation was not affected by this single acute treatment, and other NOX family members were not assessed. Therefore, we aimed to explore the effect of genetic knockout (KO) of NOX2 or acute inhibition of NOX4 with GKT137831. A moderate SCI contusion injury was performed in 3 month old NOX2 KO and wild-type (WT) mice, who received no treatment or GKT137831/vehicle 30 minutes post-injury. Motor function was assessed using the Basso Mouse Scale (BMS), followed by evaluation of inflammation and oxidative stress markers. NOX2 KO mice, but not GKT137831 treated mice, demonstrated significantly improved BMS scores at 7, 14, and 28 days post injury (DPI) in comparison to WT mice. However, both NOX2 KO and GKT137831 significantly reduced ROS production and oxidative stress markers. Furthermore, a shift in microglial activation toward a more neuroprotective, anti-inflammatory state was observed in KO mice at 7 DPI and a reduction of microglial markers at 28 days. While acute alterations in inflammation were noted with GKT137831 administration, this was not sustained through 28 days. *In vitro* analysis also showed that while GKT137831 reduced ROS production by microglia, it did not translate to changes in pro-inflammatory marker expression within these cells. These data demonstrate that NOX2 and NOX4 play a role in post-injury ROS, but a single dose of NOX4 inhibitor fails to enhance long-term recovery.

## Introduction

Inflammation and oxidative stress occurring after spinal cord injury (SCI) play significant roles in post-injury recovery. An overproduction of reactive oxygen species (ROS) contributes to post-injury oxidative stress and is observed weeks to years after injury [1]. The nicotinamide adenine dinucleotide phosphate (NADPH) oxidase (NOX) enzyme family is one of the primary synthesizers of ROS in the injured spinal cord [2, 3]. We and others have shown that the NOX2 and 4 isoforms are up-regulated after brain and SCI in a number of cells [3–5].

In microglia after mouse SCI, NOX2 expression was elevated at 4 and 7 DPI, while NOX4 expression was significantly elevated at 1, 4 and 60 DPI [6]. Inhibition of NOX2, either indirectly with a non-specific inhibitor such as apocynin or via NOX2 knockout (KO), has been shown to reduce acute oxidative stress and improve acute recovery in traumatic brain injury (TBI) models [7]. Also in the brain injury model, delayed administration of gp91ds-tat, which inhibits NOX2 assembly, induced a shift in microglial polarization from pro-inflammatory phenotype to the anti-inflammatory phenotype [8]. We have previously shown that acute inhibition of NOX2 with this same treatment had similar effects in a SCI model, with elevated microglial anti-inflammatory marker expression at subacute periods after moderate SCI in mice [9]. In addition, in this same study acute gp91ds-tat administration reduced acute and sub-acute post-injury inflammation, ROS production and improved motor function after SCI, but chronic inflammation in the injured spinal cord tissue was not found to be altered. Recently, Sabirzhanov et al. [5] demonstrated similar results with the NOX2 KO model, with sustained improvements in motor function that was similar to gp91ds-tat administration accompanied by suppressed thermal and mechanical hypersensitivity. This evaluation in NOX2 KO mice also demonstrated sustained white matter sparing at 8 weeks post-injury, as well as acute depression in macrophage infiltration at 24 hours, but sub-acute evaluation was limited.

To date, the contribution of NOX4 to recovery after SCI is unclear. NOX4, which is also upregulated after SCI [3, 4], differs from other NOX isoforms in several ways. This enzyme produces hydrogen peroxide instead of superoxide and is constitutively active independent of cytosolic subunits [10]. This isoform has been reported to be largely regulated on the transcriptional level, however post-transcriptional regulation has also been reported [11, 12]. Originally found in the kidney and named Renox [13], NOX4 has since been described to have a very wide tissue distribution and to be highly expressed in the epithelium, including within brain vessels [14]. In the rat brain, NOX4 was found to be expressed in microglia and astrocytes after injury, and constitutively expressed in neurons [4]. In the healthy rat spinal cord, NOX4 was not found in motor neurons, but was expressed in both astrocytes and microglia, with an elevation in expression acutely after injury in microglia [3, 15]. Clinically, after TBI, Li et al. [16] found an acute peak in NOX4 in neurons and a gradual increase in astrocytes that continued until the end of the study, although expression in microglia was not examined. Interestingly, the authors found this increased immunostaining of NOX4 to be correlated with a poorer condition of patients, as measured by the Glasgow Coma Score (GCS).

The role of acute NOX4 expression and the influence of NOX2 knockout during the sub-acute period after SCI, in comparison to our previous work on acute NOX2 inhibition with gp91ds-tat, remains a gap in the SCI research field. This study therefore aimed to investigate the effect of acute inhibition of NOX4 or complete elimination of NOX2 activity through utilization of the NOX4 inhibitor GKT137831 or the NOX2 KO mouse model. While GKT137831 is not specific for NOX4, and may also target NOX1, NOX1 has limited expression within the rodent spinal cord, suggesting effects may be specific for NOX4 [17]. We now show that global genetic ablation of NOX2 has very similar effects as a single acute administration of a NOX2 inhibitor, but that a single dose of a NOX4 inhibitor is less effective. While both NOX2 and NOX4 inhibition reduced acute and sub-acute oxidative stress and inflammation, only NOX2 inhibition led to persistent reductions and functional recovery.

## Material and methods

### Experimental design

Adult male NOX2 KO (20-25g, B6.129S-*Cybb*^*tm1Din*^/J, Jackson Laboratories, knock out of cytochrome b-245, beta polypeptide, C57Bl6 background) and wild type (WT, C57Bl6, 20-25g, Taconic Farms, Derwood, MD) mice were utilized in all experiments (Table 1). Groups including KO and WT mice, as well as WT mice that received vehicle or GKT137831. Mice underwent a moderate spinal cord injury contusion at T9, followed by weekly motor assessment using the BMS score. NOX2 KO and WT mice then underwent sub-acute assessment of oxidative stress and inflammatory responses, including detection of protein carbonylation with Oxyblot, pro- and anti-inflammatory marker expression with Western blot, microglial presence and neuronal survival with immunohistochemistry. WT mice administered vehicle or GKT137831 underwent acute and sub-acute assessment of similar outcomes, including detection of acute ROS with CM-H2DCFDA assay, protein carbonylation, and inflammatory cell presence at the lesion site with immunohistochemistry and flow cytometry. In addition, as the results of the GKT137831 study were somewhat surprising, determination of the effect of GKT137831 was evaluated *in vitro* on isolated microglial cells or the microglial cell line BV2. All outcome measures and n for each experiment are detailed in Table 1.

**Table 1:** Population and experimental groups per assay throughout the experimental design. Please note that due to the experimental design, in which treatment studies were run in series rather than parallel, different outcome measures were utilized for NOX2 KO experiments in comparison to GKT137831 experiments. To help clarify animal and cell culture utilization, n for each outcome measure and time point is provided.

Outcome Measure	BMS (weekly)	Oxyblot/Western blot	CM-H2DCFDA	Immunohisto-chemistry	Flow Cytometry	Cell based Assays
WT Mice	10	4 (7DPI), 6 (28DPI)		3 (28DPI)		
NOX2 KO Mice	12	4 (7DPI), 6 (28DPI)		4 (28DPI)		
Vehicle Treated Mice	6 (1 mouse removed)	4 (28DPI)	4		6 (7DPI)	
GKT137831 Treated Mice	9 (1 mouse removed)	4 (28DPI)	4		7 (7DPI) (1 mouse removed)	
In vitro—GKT137831/Vehicle					3 biological replicates	3 biological replicates

*Note–animals who were removed from the study prior to 28 days were not included in BMS testing. When animals were removed from the study, the n remaining is the final n included in the study.

### Animal handling and surgical methods

Mice were group housed (4–5 mice per cage) and received free access to food and water with a 12:12 hour light cycle. A total of 82 male mice (NOX2 KO n = 16, WT n = 53, WT naïve n = 3) were used for this study and randomly assigned to experimental groups; due to post-surgical complications, 3 mice (1 WT, 2 WT + GKT137831; removals indicated in Table 1) were removed from the study. Animal numbers were established prior to the study using power analysis based on prior research for BMS scoring, histology and flow cytometry to achieve a power of 80% with an alpha of 0.05. Investigators were blinded to treatment and genetic grouping. All experiments complied fully with the principles set forth in the “Guide for the Care and Use of Laboratory Animals” and were approved by the Uniformed Services University IACUC.

For SCI, mice were anesthetized with isoflurane (4% induction, 2% maintenance). Mice received a laminectomy at the T9 spinal level, followed by a contusion simulating moderate SCI using the Infinite Horizons Impactor (50 kdyne; Precision Systems and Instrumentation, Fairfax Station, VA) as previously described [9]. The incision was then closed and animals were maintained on heating pads until they regained movement. Acetaminophen (Children’s Tylenol, 200mg/kg) was added to drinking water for 72 hours post-injury. Manual bladder expression was performed daily until normal bladder expression returned.

### GKT137831 administration

At 30 minutes post-injury, either GKT137831 or vehicle (1.2% methyl cellulose (Sigma, M0262-100g) and 0.1% polysorbate 80 (Sigma, 59924-100G-F) per 100mL de-ionized water) was administered by oral gavage to a cohort of WT mice. GKT137831 was obtained from Genkyotex (Geneva, Switzerland), and reconstituted for a final dose of 60mg/kg and volume of 10ml/kg.

### Functional testing

Locomotor function and recovery was analyzed using the Basso Mouse Scale for Locomotion (BMS) and subscore by investigators blinded to group, as previously described [18]. Injured mice were scored in seven categories including ankle movement, plantar placement, stepping, coordination, paw position, trunk instability and tail position. BMS subscore allows for analysis of paw position, trunk stability and coordination, once mice achieve the threshold of frequent stepping [18], All mice were scored at 24hr post injury and weekly thereafter.

### Oxyblot

At 7 or 28 days post injury (DPI), mice were euthanized and tissue was flushed with 100ml of 0.9% Sodium Chloride. A 5mm spinal cord segment, 2.5mm rostral and 2.5mm caudal to the lesion epicenter, was collected and protein extracted with RIPA (1X) buffer and Halt protease inhibitor single-use cocktail (Thermo Scientific, Rockford, IL). Millipore OxyBlot Protein Oxidation Detection Kit was used according to the manufacturer’s instructions. GAPDH (1:2000, MAB374, Millipore) was used as control for gel loading and protein transfer. Bands were quantified using the Image J software.

### CM-H2DCFDA staining

At 2 hours post-injury, WT-vehicle or GKT137831 treated mice were euthanized with euthasol (sodium pentobarbital mix, 200mg/kg) and spinal cords were rapidly dissected and frozen on dry ice. Fresh frozen spinal cords (10 mm centered on the T9 lesion site) were cut longitudinally with a cryostat and every 3^rd^ 20 μm slice mounted onto charged slides. ROS level in tissue was assessed using fluorescence probe 2’,7’-dichlorodihydrofluorescein diacetate (H2DCFDA; Molecular Probes) as previously described [19]. CM-H2DCFDA is hydrolyzed by nonspecific esterases to release 2′,7′-dichlorodihydrofluorescein (CM-H2DCF), which is oxidized by intracellular ROS, such as hydrogen peroxide, to CM-DCF, which emits a green fluorescence. Briefly, slides were washed with chilled PBS for 5 minutes then incubated at 37°C in a humid chamber covered with 500 μl of 5μM H2DCFDA diluted in PBS. After incubation a last wash with PBS was performed and slides were coverslipped using mounting media containing DAPI (Vector Labs). Fluorescence within the lesion epicenter +/- 1mm was detected and photographed using the NanoZoomer Digital Pathology system (Hamamatsu Photonics, K.K., Japan). Scion Image Analysis was used to assess density of DCF fluorescent pixels above background. Measurement was performed on at least 5 randomly selected images from the dorsal half of the spinal cord obtained per animal in the perilesional region (the 1mm surrounding the lesion epicenter, not including the central cavity) including grey and white matter.

### Western blot

Utilizing the same tissue obtained for Oxyblot, aliquots of 25μg protein were used for western analysis using the following primary antibodies: anti-3NT (1:1000, Ab61392 Abcam, Cambridge, MA), anti-CD86 (1:250, Ab53004 Abcam), anti-iNOS (1:1000, Ab3523 Abcam), anti-GFAP (1:100, Ab4648 Abcam), and anti-CD11b (1:10,000; MCA275R Abd Serotec). Immune complexes were detected with appropriate secondary antibodies and chemiluminescence reagents (Pierce, Rockford, IL). All samples were normalized to GAPDH (1:2000, Millipore, Temecula, CA). ImageJ software was used to quantify bands.

### Immunohistochemistry

At 28 DPI, tissue was assessed for immunohistochemistry. Mice were anesthetized (Euthasol, 0.22ml/kg, I.P) and perfused with 100ml of 0.9% sterile saline, followed by 300ml of 10% buffered formalin phosphate (Fisher Scientific, Fair Lawn, NJ). A 5mm spinal cord segment, 2.5mm caudal and 2.5mm rostral to the injury site, was extracted and kept in formalin for 24 hours and then transferred to a 30% sucrose solution. Spinal cords were then cut into 20μm axial sections. Five sections spanning the lesion site (rostral 2.5mm to caudal 2.5 mm) at regular intervals (every 1mm) were selected for standard fluorescent immunohistochemistry utilizing primary antibodies that had been previously characterized (using lack of primary antibody or blocking antibody and examination of expected cellular morphology and staining profiles) in the laboratory [3, 4], including Iba1 (1:100, 019–19741 Wako), and NeuN (1:100, ABN78 Millipore). Alexa-Fluor secondary antibodies (1:1000, A11010, A11070 or A11017 Invitrogen) were used for visualization. Slides were coverslipped using mounting media containing DAPI to counterstain for nuclei (Vector Labs, Burlingame, CA). Immunofluorescence was detected and photographed in the dorsal column region within the 5mm region of interest using an Olympus DP72 microscope with Olympus cellSens microscopy software (Olympus, Center Valley, PA). NeuN was quantified using ImageJ, using first image threshold to identify only cells positively stained with NeuN, followed by the particle analysis tool, with a cutoff of 500 pixels. All other immunolabeled cells were quantified using threshold analysis and pixel density above threshold with ImageJ as previously described [20].

### Flow cytometry

A 5mm spinal cord segment, 2.5mm caudal and 2.5mm rostral to the injury site, was processed for flow cytometry at 7 days post-injury (DPI), as previously described [9]. Briefly, the OptiPrep density gradient was used to exclude the debris layer from the viable cells. Sytox Blue (Thermo Scientific, Rockford, IL) was used to gate out dead cells. APC/Cy7 conjugated anti-CD45 (1:50, BioLegend, San Diego, CA) was used to gate on population of interest. From there, populations were further gated on microglia/macrophages (CD45^+^ CD11b^+^ GR-1^**-**^), and neutrophils (CD45^+^ CD11b^+^ GR-1^+^) using PE conjugated anti-CD11b (1:20 eBioscience Inc., San Diego, CA) and FITC conjugated anti-GR-1 (1:200 BioLegend, San Diego, CA). All analysis was performed using FlowJo (FlowJo, LLC, Ashland, OR). OneComp eBeads (one drop per sample, eBioscience Inc., San Diego, CA) were used for single stain controls, while cells were used for unstained control. Further statistical analysis was performed on values/ percentages gathered. Each animal represents one sample with a minimum of 200,000 cells collected.

BV2 microglia (described below) were detached from culture plates with Accutase^®^ cell detachment solution (Innovative Cell Technologies, San Diego, CA). Cells were immunolabeled with PE conjugated anti-CD86 (0.5 μl/ml, BD Biosciences, San Jose, CA, AF647 CD206 (1μl/ml, BD Biosciences, San Jose, CA), BV421 conjugated anti-TGFβ (2 μl/ml, BD Biosciences, San Jose, CA), and Fixable Viability Stain 510 (0.5 μl/ml, BD Biosciences, San Jose, CA).

### *In vitro* analysis

The BV2 microglial cell line (a generous gift from Dr. Carol Colton) was cultured and replated at passage 13–20. Cells were incubated at 37°C with 5% CO_2_ in Dulbecco’s modified Eagle media (Gibco, Carlsbad, CA) with 10% fetal calf serum (Hyclone, Logan, UT), 1% L-glutamine (Gibco), 1% sodium pyruvate (Gibco), and 1% Pen/Strep (Fisher, Pittsburgh, PA). Cells were used 24 hours after plating for experimentation at a density of 2 x10^5^ cells/ml. BV2 microglia were exposed to LPS (100ng/ml) or vehicle for 1 hour prior to treatment with GKT137831 (0.5μM, 1μM, 5μM, 10μM) or vehicle and incubated at 37°C and 5% CO_2_ for 24 hours. All drugs were prepared and stored according to the manufacturer’s guidelines.

Primary microglia were obtained as previously described [15]. Briefly, the whole brain was dissected from P2 Sprague Dawley rat pups and homogenized in L15 media (Gibco). Mixed glial cultures were then incubated for 8 to 10 days at 37°C with 5% CO2 in Dulbecco’s modified Eagle media (Invitrogen) with 10% fetal calf serum (Hyclone, Logan, UT, USA), 1% L-glutamine (Invitrogen), 1% sodium pyruvate (Invitrogen), and 1% Pen/Strep (Fisher, Pittsburgh, PA, USA). After the initial incubation, cells were shaken for 1 hour at 100 rpm and 37°C and detached microglia were collected and plated at 2 x10^5^.

Twenty-four hours after plating, microglia were exposed to LPS (100ng/ml) or vehicle for 1 hour prior to addition of GKT137831 (10μM) or vehicle and incubation at 37°C and 5% CO_2_ for 24 hours. All assays were performed in 3 independent trials each in triplicate.

### Nitric oxide and ROS (DCF) assay

Nitric oxide production and intracellular ROS generation were determined 24 hours after treatment. NO^•^ production was assayed using the Griess Reagent Assay kit (Invitrogen) and absorption read at 540nm, according to the manufacturer’s instructions. The intracellular levels of ROS were measured using the fluorescence probe 2’,7’-dichlorodihydrofluorescein diacetate (H2DCFDA; Molecular Probes, Eugene, OR). Cells were incubated with 10 μM H2DCFDA (diluted in PBS) for 45 min at 37°C. Fluorescence was measured using excitation and emission wavelengths of 490 and 535 nm, respectively, according to the manufacturer’s instructions.

### ELISA

At 24 hours after treatment, 150 μl of media was transferred to new plates and frozen at -80°C. In order to assess the changes in cytokine release after treatment with GKT137831, ELISA was used to determine IL-1β (Thermo Fisher) and TNFα (Millipore) release. All assays were performed as per manufacturers’ instructions.

### Statistics

Quantitative data are presented as mean ± standard error of the mean. Normality of data was assessed using the Shapiro-Wilk test within GraphPad Prism. BMS and subscore were analyzed using repeated measures ANOVA with Bonferroni’s multiple comparisons test. All other quantitative data were analyzed using paired *t* test, unpaired *t* test, one-way or two-way ANOVA, as appropriate. All statistical tests were performed using the GraphPad Prism Program, Version 9 for Windows (GraphPad Software, San Diego, CA). A *p* value <0.05 was considered statistically significant.

## Results

### NOX2 KO, but not acute NOX4 inhibition, improves functional recovery

**Fig 1:**
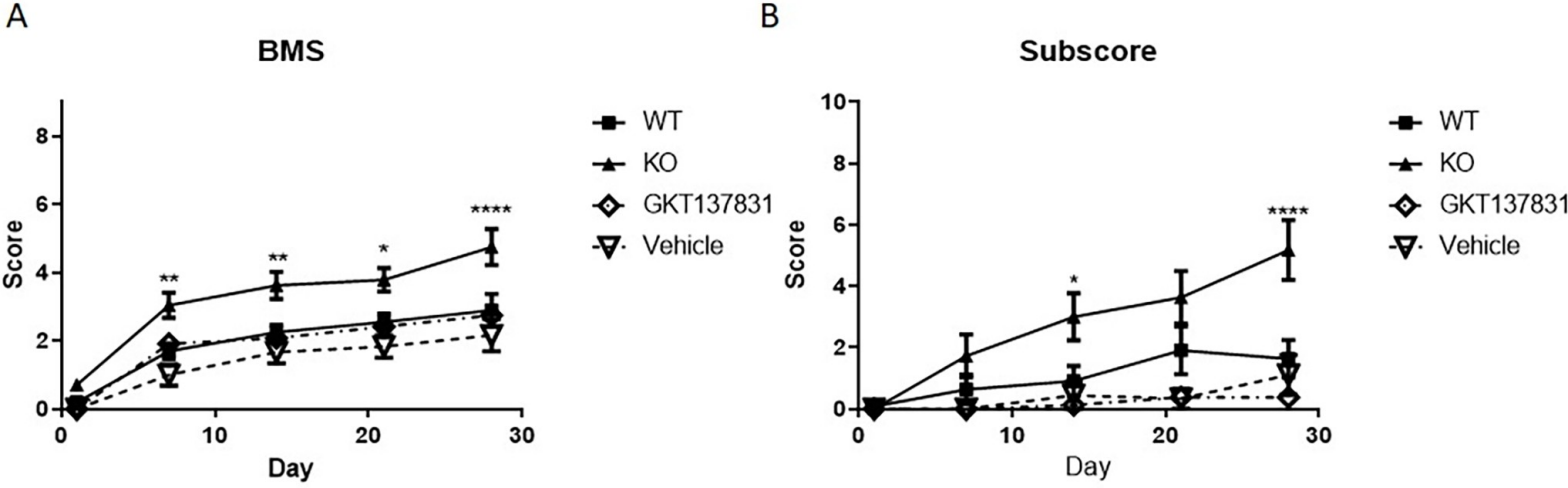
Hind limb locomotion recovery post surgery was measured using Basso Mouse Scale (BMS) at 1, 7, 14, 21, and 28 days post injury. The BMS average score showed significant improvement at 7, 14, 21 and 28 days in NOX2 KO injured mice compared to wild type injured mice (A). Subscore analysis showed significant improvement at 28 days post-injury in the KO group (B). Points represent mean +/- SEM., NOX2 KO injured n = 12, WT injured n = 10, Vehicle n = 6, GKT n = 9. *p<0.05, **p<0.01, ****p<0.0001 repeated measures ANOVA.

Hind limb locomotion recovery post-surgery was measured using the BMS at 1 day and weekly thereafter through 28 DPI (Fig 1). No significant difference in function was found at 1 day post-injury between any group, demonstrating a consistent level of injury among groups. However, the BMS score showed significant (p = 0.0042 WT vs KO over time, two-way ANOVA) increases in score in the NOX2 KO mice compared to WT injured mice at 7 (1.70 +/- 0.2 vs. 3.04+/-0.4), 14 (2.25+/-0.2 vs 3.63+/-0.4), and 28 DPI (2.9+/-0.5 vs. 4.75+/-0.5), suggesting that NOX2 KO improves gross motor function, similar to acute post-injury administration of gp91ds-tat [9]. In addition, the BMS subscore, which evaluates the stepping behavior of the mouse, including frequency of plantar stepping and coordination and paw position during steps, showed a significant improvement with NOX2 KO by 28 days post-injury (p = 0.0187 WT vs KO day 28, repeated measures ANOVA).

In contrast, GKT137831 and vehicle treated mice showed no significant difference between groups or in comparison to the WT (p = 0.4156, repeated measures ANOVA). Similarly, no significant difference between groups was noted in the BMS sub-score (p = 0.1867 Vehicle vs. GKT137831 day 28, repeated measures ANOVA, Fig 1), suggesting that, unlike gp91ds-tat targeting of NOX2, a single administration of NOX4 inhibitor does not markedly change motor function.

### NOX2 KO, but not NOX4 inhibition, reduces oxidative stress after SCI

**Fig 2:**
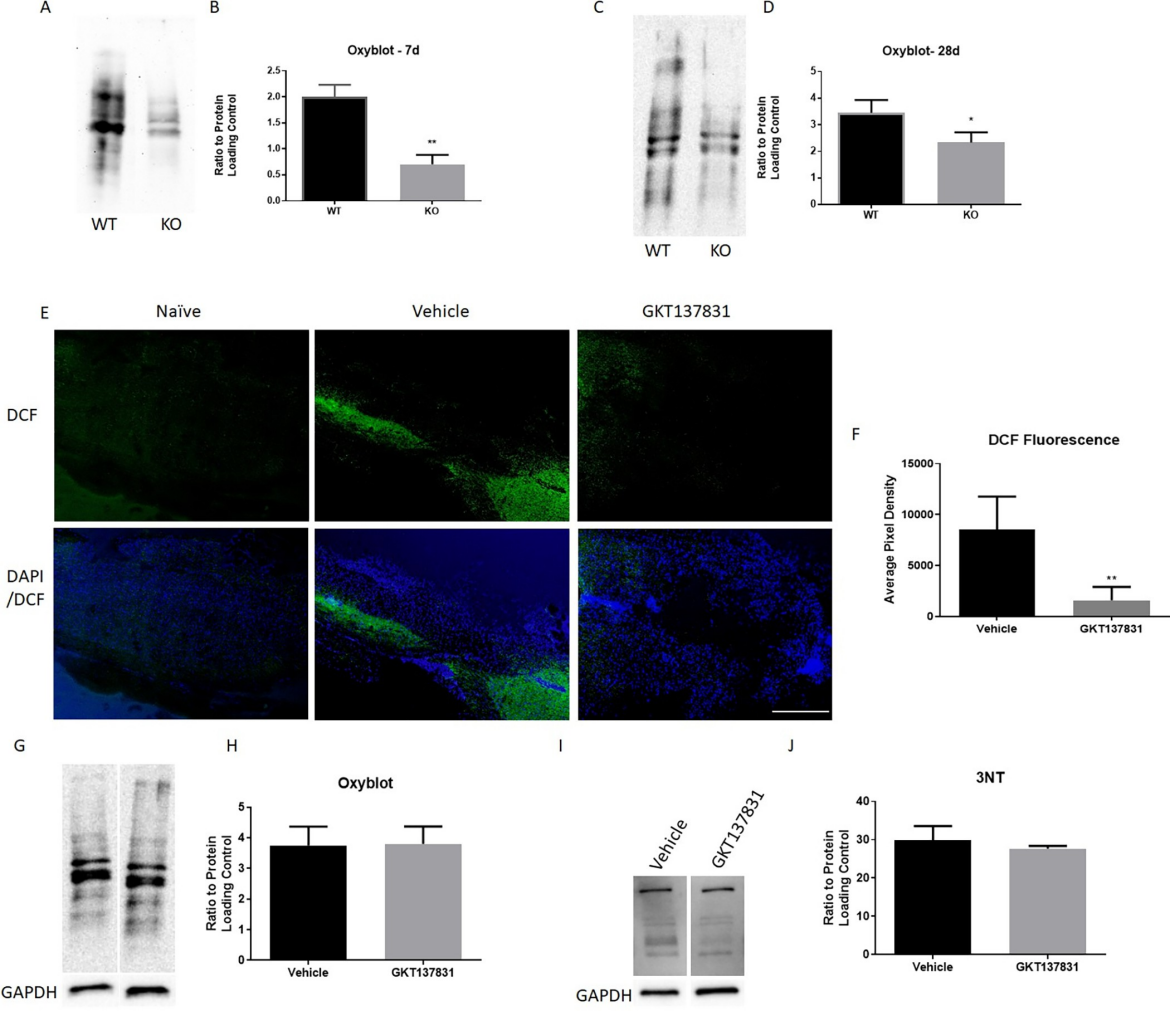
NOX2 knockout and NOX4 inhibition offer acute inhibition of oxidative stress, but only NOX2 knockout reduces chronic post-injury oxidative stress. Oxyblot was used to measure oxidative stress in tissue surrounding the lesion site at 7 (A, B) and 28 days (C, D) in NOX2 WT and KO mice. Representative blots (A, C) and quantitation (B, D) are shown. GAPDH was used to normalize data. CM-H2DCFDA (DCF) staining was performed on tissue at 2 hours after injury to assess acute ROS production with GKT137831 or vehicle treatment. GKT137831 reduced ROS as shown by reduced green DCF fluorescence at the lesion site (lesion epicenter at T9 at the center of the image) compared to vehicle control (E). Pixel density quantification showed this is a significant reduction in DCF fluorescence on GKT137831 treated tissue (F). However, no other marker of oxidative stress was assessed acutely after injury, and oxyblot and 3NT western blotting performed on 28 DPI tissue lysates, shown in (G) and (I), respectively, do not show significant change with treatment. Oxyblot showed protein carbonylation did not significantly differ between vehicle and GKT137831 groups at 28 days (H). 3NT blotting also showed nitrosylation did not significantly differ between the 2 groups (J). *p<0.05, **p<0.01, N = 4/group at 7 dpi (NOX2 KO/WT) and 28dpi (GKT137831, Vehicle), N = 6/group at 28dpi (NOX2/WT). Bars represent mean +/- SEM.

To determine if the beneficial effects of NOX2 KO were associated with reductions in oxidative stress, oxidative stress markers in tissue surrounding the lesion site was measured at 7 and 28 DPI (Fig 2). Protein carbonylation was measured using the Oxyblot kit, and demonstrated that both in the sub-acute 7 day period (p<0.01, unpaired t test, Fig 2A and 2B) and the chronic 28 day period (p<0.05, unpaired t test, Fig 2C and 2D), NOX2 KO led to a significant reduction in protein carbonylation in comparison to WT mice, similarly to what was previously observed with gp91ds-tat acute administration [9].

GKT137831 was not found to improve motor function, so we aimed to determine if NOX4 inhibition reduced acute ROS production in the spinal cord after injury. Examination of the conversion of CM-H2DCF to CM-DCF, indicative of ROS presence, showed an increase with injury at 2 hours (Fig 2E). GKT137831 administration was found to significantly reduce CM-DCF fluorescence (p = 0.0076, unpaired t-test; Fig 2E and 2F), demonstrating an acute reduction in ROS presence with NOX4 inhibition.

However, NOX4 inhibition failed to show a chronic reduction in oxidative stress markers that was observed in NOX2 KO or gp91ds-tat administered mice [9]. Oxyblot was used to measure the protein carbonylation between the vehicle and the GKT137831 treated groups at 28 days post-injury (Fig 2F and 2H), while 3NT was used to measure nitrosylation (Fig 2I and 2J). No significance was seen between groups for either marker (p = 0.9585, 0.5209, respectively, unpaired t test).

### NOX2 KO and NOX4 inhibition reduces sub-acute microglial activation

**Fig 3:**
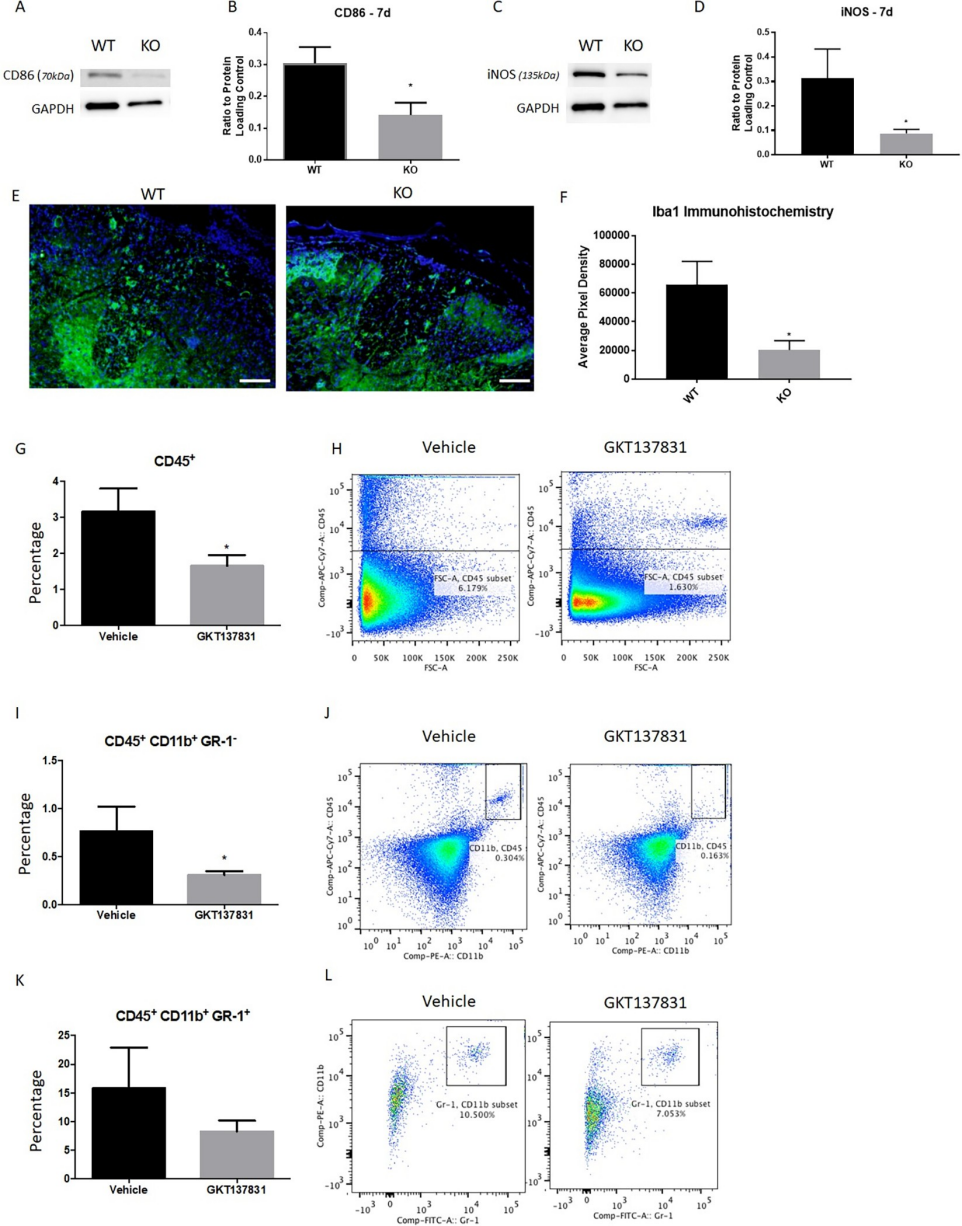
Markers of inflammation were significantly reduced at 7dpi by NOX2 and NOX4 inhibition, but remained so only with NOX2 KO at 28 DPI. Western blotting for inflammatory markers CD86 (A, B) and iNOS (C, D) were significantly altered by NOX2 KO at 7dpi. GAPDH was used to normalize data. N = 4/group. Iba1 immunohistochemistry showed a continuation of inflammation depression with significant reduction at 28 DPI as measured by threshold pixel density analysis (E, F). Images shown 1.5mm rostral to the lesion epicenter. N = 4/group. Size bar = 200μM. Flow cytometry was performed on tissue at 7 DPI to measure inflammatory cell populations in vehicle or GKT137831 treated spinal cords. All inflammatory cells were marked with CD45^+^ and showed a significant reduction with GKT137831 treatment (G, H). CD45^+^ CD11b^+^ GR-1^**-**^ were interpreted as the macrophage/microglia population and showed a significant reduction (I, J). CD45^+^ CD11b^+^ GR-1^+^ were used to identify the neutrophil population and GKT137831 induced no significant change in these cells (K, L). N = 6-7/group. *p<0.05, unpaired t-test. Bars represent mean +/- SEM.

To determine if NOX activity had an influence on microglial and macrophage activation, expression of various pro- and anti-inflammatory markers was assessed using a variety of methods. Expression of pro-inflammatory markers CD86 and iNOS showed significant reductions in expression in NOX2 KO mice in comparison to WT mice (p = 0.047, p = 0.013, respectively, unpaired t test, Fig 3A–3D). By 28 DPI, no difference was observed between NOX2 KO and WT groups in expression of these same markers (data not shown), similar to our previous findings with gp91ds-tat administration [9]. However, analysis of overall macrophage/microglia presence in the injured spinal cord showed an influence of NOX2 KO on Iba1 expression at the lesion site; quantitation of Iba1 immunoreactivity at 28 DPI showed a significant reduction in KO tissue (p = 0.034, unpaired t-test, Fig 3E and 3F), which was not observed in our prior work [9].

Flow cytometry was used to assess immune cell populations in the injured spinal cord 7 DPI with and without GKT137831 administration (Fig 3G–3L). All cells of interest were marked with CD45^+^ marker (Fig 3G and 3H). GKT137831 treated tissue showed a significant reduction in this population of CD45^+^ cells (p = 0.0241, unpaired t-test; Fig 3G) in comparison to the vehicle treated group. This reduction was most prominent in the macrophage/microglia populations, labeled as CD45+CD11b+GR1- (p = 0.0431,unpaired t-test; Fig 3I and 3J). Neutrophils (CD45+CD11b+GR1+) showed no significant change in cell population with GKT137831 administration (p = 0.2912, unpaired t-test; Fig 3K and 3L). These reductions were similar to those observed in the subacute period following single dose gp91ds-tat administration [9].

### NOX2 KO improves neuronal viability

**Fig 4:**
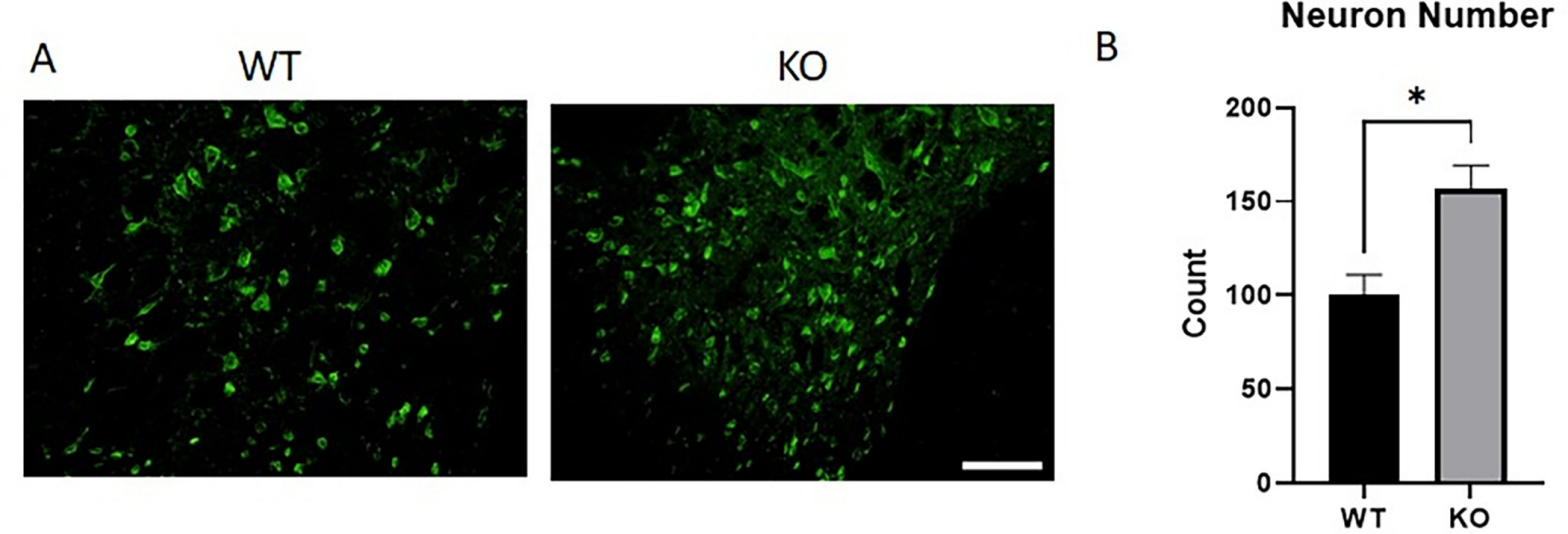
Neuronal viability in the dorsal horn adjacent to the lesion site was greater with NOX2 KO at 28 days post-injury. NeuN is a typically nuclear neuronal marker and was significantly greater in NOX2 KO injured tissue at 28 DPI. Representative images 0.5mm rostral to the lesion site are shown, and demonstrate that NeuN stain is often cytoplasmic in the WT tissue, while it is more nuclear in the KO tissue, as well as being present in a greater amount in the KO tissue. Bars represent mean +/- SEM. N = 3-4/group. *p<0.05, unpaired t test. Size bar = 200μM.

To determine if these changes in inflammation and oxidative stress resulted in neuroprotection, NeuN protein expression was assessed at 28 DPI in WT or NOX2 KO mice. In the dorsal horns, near the lesion site, NOX2 KO mice demonstrated more NeuN staining in the dorsal horns (Fig 4A). Quantitation of NeuN pixel density in the dorsal horn showed a significant decrease in WT NeuN expression in comparison to KO (p = 0.0193, unpaired t-test, Fig 4B).

### Microglia show reduced ROS expression, but not pro-inflammatory markers, in response to GKT137831

**Fig 5:**
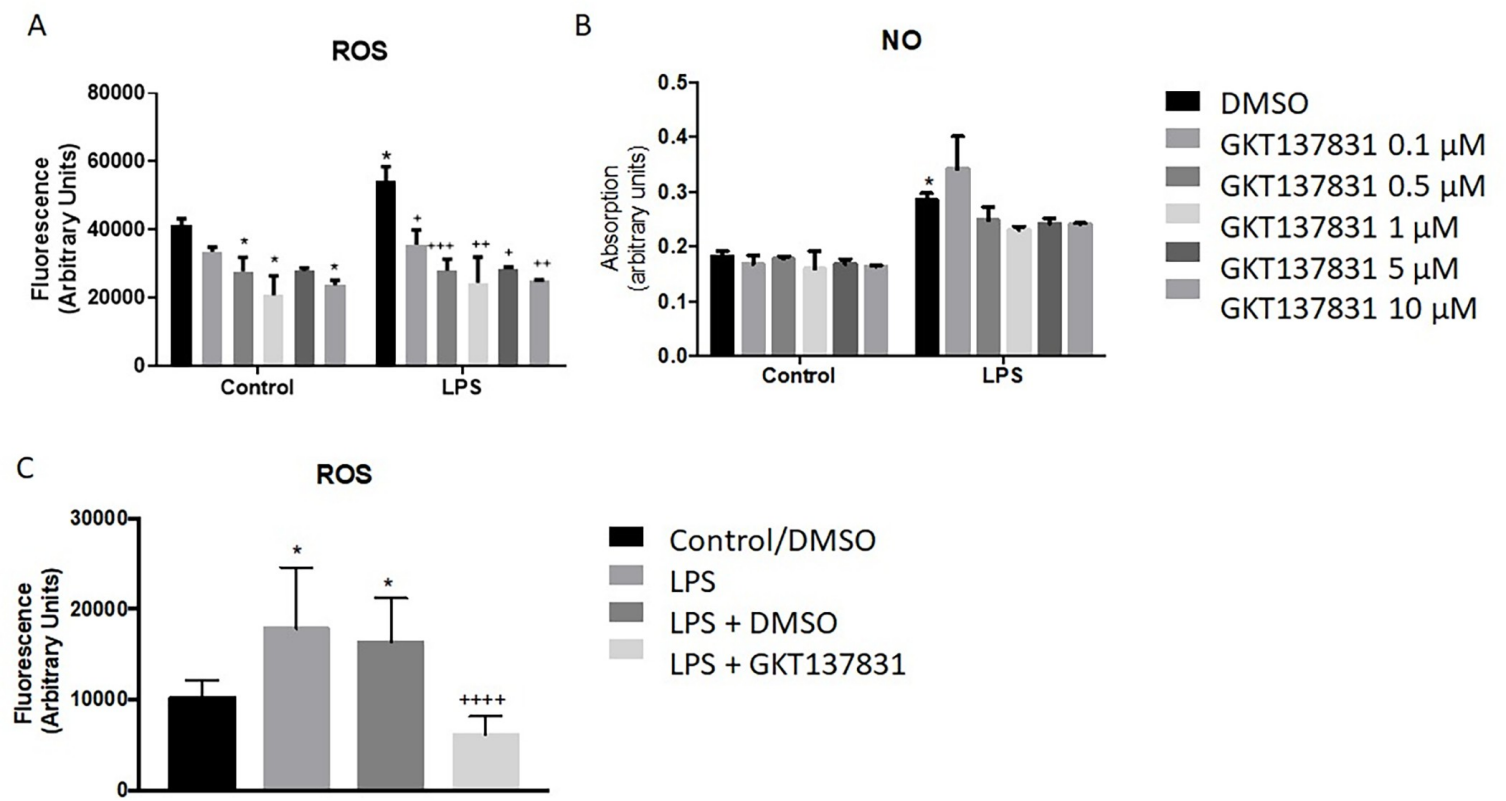
GKT137831 treatment in microglia reduces LPS induced ROS in a dose-dependent fashion, but has no effect in NO release. BV2 microglial cells were stimulated with LPS and treated with the NOX inhibitor GKT137831 one hour after stimulation. 24 hours after ROS and NO production was assessed using DCF and Greiss assay, respectively. Microglia show a dose dependent significant decrease of ROS release in GKT137831 treated compared to control or LPS alone (A). NO shows no significant reduction with any concentration of GKT137831 (B) ROS production decrease in response to GKT137831 treatment was confirmed in primary microglial cells (C). N = 3. *p<0.05 compared to control, ^+^p<0.05, ^++^p<0.01, ^+++^p<0.001 compared to LPS, using Two-Way ANOVA (A)(B) and One-Way ANOVA (C) with Dunnett’s Multiple Comparison post-test. Bars represent mean score +/- SEM.

The limited effect of NOX4 inhibition in the spinal cord of mice prompted further evaluation of the effect of GKT137831 on microglia. We utilized both the BV2 microglial cell line and a primary rat microglia cell culture stimulated with the pro-inflammatory agent LPS as *in vitro* models. ROS and nitric oxide release were assessed 24 hours after stimulation and administration of GKT137831. Baseline ROS in BV2 cells was significantly reduced by GKT137831 administration (p<0.0001, two-way ANOVA, Fig 5A); while ROS was increased by LPS, this was significantly reduced in a concentration dependent manner by GKT137831 (p = 0.0023, two-way ANOVA; Fig 5A). This effect on ROS production was further confirmed using primary microglia (overall p<0.0001, LPS-DMSO vs LPS-GKT137831 p = 0.0002, one-way ANOVA; Fig 5C). Nitric oxide release was not altered by GKT137831 administration (p = 0.6624, two-way ANOVA; Fig 5B), suggesting an incomplete reduction in microglial related inflammation with NOX4 inhibition, unlike what we have previously observed with gp91ds-tat administration in cultured microglia [4].

**Fig 6:**
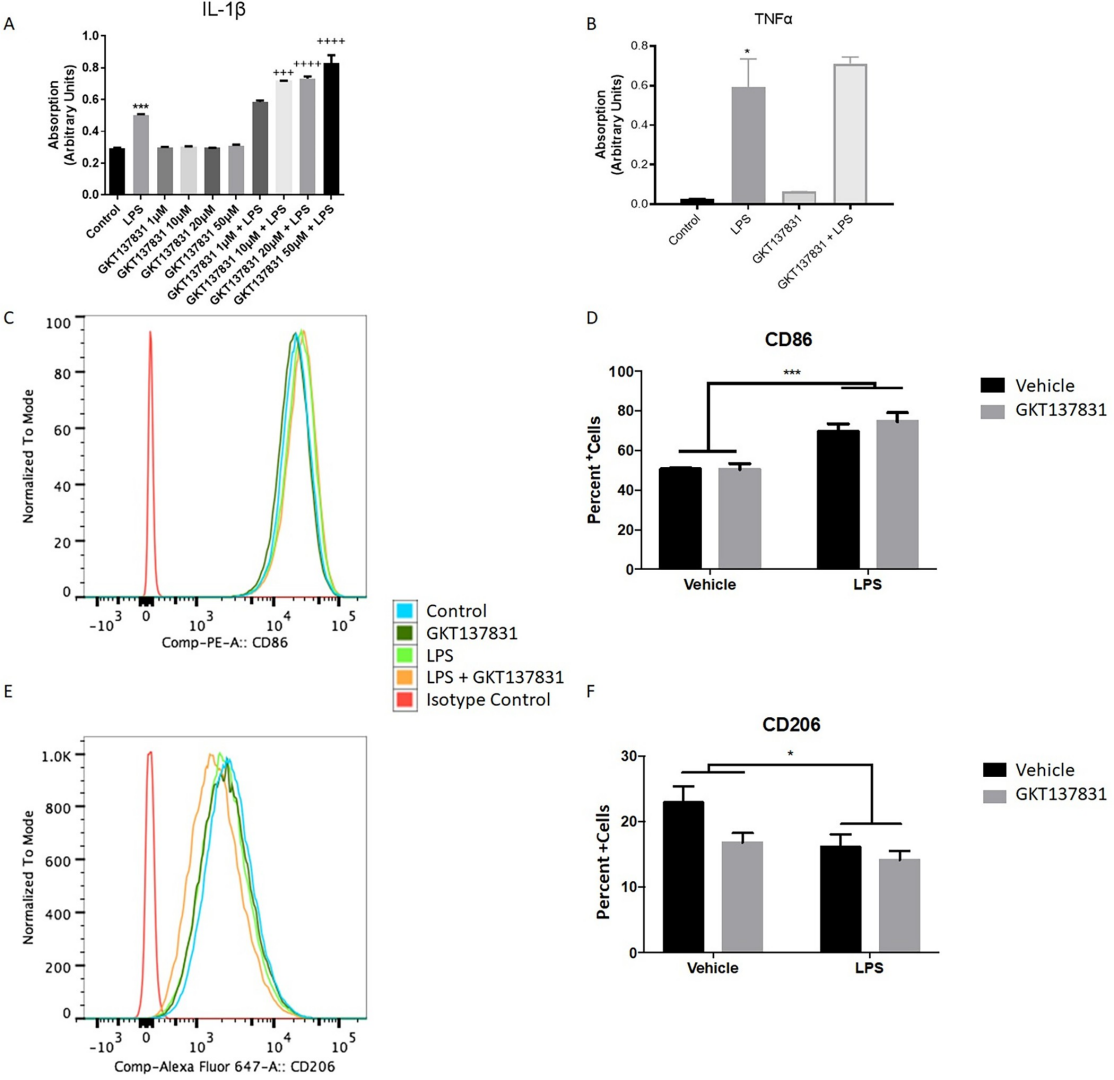
GKT137831 treatment has limited effects on microglial related inflammatory responses. BV2 microglia were stimulated with LPS and treated 1 hour after with the NOX inhibitor GKT137831 at 0.5μM, 1μM, 5μM, 10μM. Media was collected 24 hours after stimulation. IL-1β ELISA assay shows no response by microglia to GKT137831 along, but in conjunction with LPS there was a significant dose dependent increase in release (A). GKT137831 showed no effect on TNF-α release, with or without LPS stimulation (B). When BV2 microglia were stimulated with LPS, a significant increase of the pro-inflammatory marker CD86^+^ cell population (C, D) and reduction in the CD206+ cell population (E, F) was observed GKT137831 did not prevent or induce any polarization changes by itself or after LPS stimulation. N = 3. *p<0.05, **p<0.01, ***p<0.001 using two-way ANOVA. Bars represent mean +/- SEM.

To further study how microglia respond to GKT137831, the pro-inflammatory cytokines IL-1β and TNFα were examined using ELISA in the BV2 cell line (Fig 6). Surprisingly, IL-1β was induced by GKT137831 in a dose dependent fashion in LPS stimulated cells (p<0.001, one-way ANOVA; Fig 6A). On the other hand, LPS induced TNFα release was unaltered by GKT137831 (p = 0.9999, one-way ANOVA; Fig 6B), again suggesting an incomplete regulation of microglial related inflammation with NOX4 inhibition.

Finally, we assessed the effects of GKT137831 on standard markers of microglial activation using flow cytometry and the BV2 cell line. We quantified the percent of positive cells for the pro-inflammatory marker CD86 (Fig 6C and 6D) and relative fluorescence of the anti-inflammatory marker CD206 (Fig 6E and 6F). A significant increase in the CD86^+^ cell population was observed when stimulated with LPS, while the frequency of CD206^+^ cell population was significantly reduced (p = 0.033 and p = 0.0002, respectively, two-way ANOVA). GKT137831 had no effect on CD86 or CD206 expression (p = 0.4867 and 0.0615, respectively, two-way ANOVA), unlike what we have previously observed with gp91ds-tat administration [6].

## Discussion

Oxidative stress significantly contributes to the pathogenesis of secondary injury by directly damaging cell components and perpetuating the inflammatory process [1]. Our prior work and that of others has demonstrated that NOX2 and NOX4 are significantly elevated in microglia after SCI [3, 21], as well as other cells [22]. Further, inhibition of NOX2 activity reduces oxidative stress and inflammation and improves functional recovery after injury [6, 9]. However it was unclear if long-term reduction using genetic knockout of NOX2 would lead to greater improvement, thus giving important information about therapeutic potential of NOX2 inhibition, or if inhibition of other members of the NOX family, such as NOX4, may improve outcomes.

Similar to the responses seen with acute inhibition, KO of NOX2 reduced ROS acutely and improved motor function. There was also a reduction in inflammation, with an increase in anti-inflammatory markers and a reduction in pro-inflammatory markers. Unlike acute inhibition of NOX2, NOX2 KO led to a chronic reduction in microglia/macrophage number at 28 DPI. However, the overall improvement in locomotor function does not appear to be different between the NOX2 KO and acute inhibition groups [9]. Very similar results have been recently shown by Sabirzhanov et al. [5], in which similar motor function improvements and neuronal survival and reduced ROS at 8 weeks post-injury were observed after SCI in NOX2 KO mice. In addition, they found that at 24 hours after injury, NOX2 KO mice demonstrated reductions in macrophage within the injury site, with accompanying very acute changes in pro- and anti-inflammatory markers. We have extended and complemented this work, by showing the same effects of NOX2 KO at the intermediate 7 day and 28 day time points.

In the mouse spinal cord injury model, protein expression of NOX2 peaks at 1–4 days post-injury [6]. It is therefore unsurprising that an acute treatment targeting this early peak would have similar effects to knockout of the gene. In the rat model, however, this expression is more extended, with elevated expression of NOX2 component gene and protein through 6 months post-injury [15, 23]. Therefore, delayed treatment approaches for NOX2 inhibition may be more effective in the rat model than mouse model. Human NOX2 expression after SCI is currently unknown, although after brain injury NOX2 is reported to be elevated acutely [16]; future work is therefore necessary to more clearly define the therapeutic potential of NOX2 inhibition.

NOX2 is directly responsible for producing ROS, particularly after injury [2]. ROS mediate clearance of debris and removal of pathogens, but can also contribute to damage to surrounding healthy tissue by elevating oxidative stress. *In vitro*, inhibition of NOX2 in microglia significantly reduces ROS production in response to LPS or other stimuli [4, 24]. Acute inhibition of NOX2 *in vivo* was previously shown to reduce oxidative stress acutely after SCI [9]; KO of NOX2 leads to an extension of that reduction. Oxidative stress can contribute to elevated inflammation and neuronal damage; amelioration of this effect of NOX2 may be a factor in the observed reductions in inflammation and neuronal loss, particularly at 28 days post-injury. These reductions were not observed in the acute inhibition study [9]. Indirect inhibition of NOX2 induced ROS, through knockout of the Hv1 extracellular proton channel, also significantly improves motor function and reduces post-injury histopathology [25].

ROS contribute to propagating inflammation. Studies have shown that ROS and elevations in oxidative stress signal, possibly through the inflammasome, to increase pro-inflammatory cytokine production [26]. NOX2 inhibition has been shown to significantly reduce sub-acute microglial numbers [9]. Interestingly, NOX2 KO was also observed to change pro- vs. anti-inflammatory marker expression, and did reduce overall number by 28 days post-injury. Previous work has suggested that activation state, not number, is more likely to influence outcome after injury [27].

We and others have previously demonstrated that NOX2 inhibition alters pro- and anti-inflammatory properties of microglia, pushing them toward an anti-inflammatory phenotype and reducing pro-inflammatory marker expression [6, 8, 28]. Following controlled cortical impact injury, NOX2 inhibition with apocynin (from 1–4 days post-injury) and NOX2 KO both effectively reduced microglial activation and pushed microglia/macrophages toward an anti-inflammatory phenotype, possibly via an NFκB mechanism. We now show additional support for this, as NOX2 KO reduces pro-inflammatory markers and increases anti-inflammatory markers at 7 days post-injury, similarly to NOX2 inhibition [9].

A number of studies have shown that inhibition of NOX2 can improve recovery after central nervous system injury. In TBI models, acute NOX2 inhibition reduced cognitive impairment and reduced lesion volume [28–30], although Kumar et al. found that inhibition of NOX2 from 1 to 3 days post-injury was not as effective at improving post-TBI function as NOX2 KO. Our current study somewhat agrees with this finding, demonstrating that NOX2 KO results in similar functional outcomes as acute NOX2 inhibition [9] in a mouse SCI model.

In contrast, while the NOX4 inhibitor GKT137831 reduced acute oxidative stress and inflammation after moderate SCI and reduced ROS production by microglia *in vitro*, this did not translate into sustained reduction in oxidative stress or a significant improvement in motor function. *In vitro*, GKT137831 has been shown to reduce ROS production by microglia in response to LPS and other stimuli, similar to the current work [24]. However, GKT137831 did not alter microglial pro-inflammatory activation. This lack of functional effect and disparate results between *in vivo* and *in vitro* experiments highlights the complexity of SCI and the role of NOX enzymes in post-injury inflammation and oxidative stress.

Oxidative stress markers after SCI can be detected within hours of injury, later peaking at acute time points and remaining present for weeks to months [1, 31]. We observed a significant reduction of oxidative stress markers at 2h in mice treated with GKT137831. This initial acute reduction in ROS release in injured tissue was consistent with our *in vitro* results in the microglial BV2 cell line and primary microglia, as well as the literature regarding NOX inhibition through GKT137831 [24, 32]. Similarly, administration of a ketogenic diet, which was found to reduce NOX4 expression and activity, potentially via inhibition of NADPH availability via the pentose phosphate shunt or by reducing HDAC function and gene expression, showed a reduction in acute and sub-acute oxidative stress [21]. As GKT137831 has shown no ROS scavenger or antioxidant activity [33], it is most likely that this reduction is a direct result of NOX inhibition in the lesion site. However, by 28 DPI, markers of oxidative stress did not show any reduction with the GKT137831 treatment, suggesting that a single acute administration does not have long-lasting effects. In contrast, a single acute administration of the NOX2 inhibitor gp91ds-tat did demonstrate long-term reductions in oxidative stress, suggesting a differential effect of the 2 isoforms in the acute period after injury [9]. Further, we have previously observed an increase in NOX4 expression at 1 to 7 days after mouse SCI [6]. Therefore, the single acute administration may not have been timed to most appropriately address the NOX4 elevation. Future work may consider delayed or repeated administration.

GKT137831 has good oral bioavailability and high plasma concentrations *in vivo* and is the most specific NOX4 inhibitor available with a very low affinity for NOX2 [32]. A single acute oral administration of the NOX4 inhibitor GKT137831 significantly reduced several inflammatory markers after a moderate SCI, including CD45^+^ cells populations at 7 DPI. However, *in vitro*, GKT137831 treatment did not lead to any changes in microglial polarization response after LPS stimulation. Furthermore, GKT137831 led to an increase in IL-1β release in LPS stimulated microglia in a dose dependent manner, but had no effect on TNF-α release. The mechanism by which IL-1β and TNFα are released by microglia in inflammatory conditions is fundamentally different; the increase in IL-1β but no change in TNFα may be related to this difference in expression properties [34]. Regardless, an increase in IL-1β was unexpected, and additional research into why this cytokine is elevated in cell culture is necessary. In a model of hypoxia-induced retinopathy, daily subcutaneous GKT137831 administration significantly reduced inflammatory mediators; this was also observed in cultured retinal microglia when GKT137831 was present during hypoxia [35]. Importantly, they also found reduced leukocyte infiltration. In agreement with these findings, genetic deletion or pharmacologic inhibition of NOX4 provided renal protection by reducing macrophage infiltration in the diabetic kidney [36]. This is consistent with our findings of a reduced monocyte population after SCI.

There is a high expression of NOX4 in endothelial cells [37]. Furthermore, NOX4 is highly present and active in the neurovasculature [38]. In a NOX4 KO model of stroke, smaller infarct volumes and neuroprotection were observed along with a reduction in the disruption of the blood–brain barrier, although inflammatory cell infiltration was not assessed [39]. NOX4 is also expressed and up-regulated in pericytes acutely after brain ischemia and its upregulation seems to enhance blood-brain barrier breakdown [40]. Furthermore, NOX4 was found to be predominant among the NOX family in human brain pericytes [14]. NOX1 has been suggested to also play a role in the vascular tissue, its expression is induced by IL-1β enhancing monocyte adhesion, while NOX4 seems to be down regulated by IL-1β in vascular cells, authors also suggest that NOX1-derived superoxide, but not hydrogen peroxide, mediates monocyte adhesion to vascular tissue [41]. All of this indicates that the acute GKT137831 administration may also be affecting vascular response in our model in agreement with the reduced inflammatory cell population found in GKT137831 treated mice. The precise mechanisms and the NOX1 and NOX4 interplay in this vascular response remain to be elucidated.

A limitation of this study is that GKT137831 was developed as an inhibitor to both NOX1 and NOX4. With comparable K_i_ values (140±40 nM for NOX4, 110±30 nM for NOX1), it is likely that NOX1 activity would also be affected after GKT137831 administration. Therefore, additional research is needed to isolate the effect of NOX1 versus NOX4. In addition, the current study only evaluated responses in male rodents; limited information is available to understand the influence of sex on NOX2 or 4 expression or impact in the injured spinal cord. To date, one study has suggested that NOX2 expression is more highly expressed in females after SCI [42].

In conclusion, NOX2 and NOX4 inhibition reduced acute oxidative stress and exerted anti-inflammatory effects in the injured spinal cord. However, these anti-inflammatory effects are not fully mediated through microglia with NOX4 inhibition, and a single treatment does not have long-lasting effects or exert significant functional benefits as single treatment was found to do with NOX2 [9]. Future work is needed to clarify the target cells of GKT137831 and to determine if repeated administration will have beneficial outcomes. In contrast, NOX2 inhibition via genetic knockout was found to be very similar to pharmacological inhibition demonstrated in our previous work [9], suggesting that targeting NOX2 after SCI may have therapeutic benefits.
